# Impact of out of pocket payments on financial risk protection indicators in a setting with no user fees: the case of Mauritius

**DOI:** 10.1186/s12939-019-0959-5

**Published:** 2019-05-03

**Authors:** Ajoy Nundoochan, Yusuf Thorabally, Sooneeraz Monohur, Justine Hsu

**Affiliations:** 1 World Health Organization Country Office, Port Louis, Mauritius; 2 Statistics Mauritius, Port Louis, Mauritius; 3 Ministry of Health and Quality of Life, Port Louis, Mauritius; 4 Department of Health Systems Governance and Financing, World Health Organization, Geneva, Switzerland

**Keywords:** Catastrophic health expenditure, Impoverishment, Out-of-pocket payments

## Abstract

**Background:**

Mauritius embraces principles of a welfare state with free health care at point of use in any public facilities. However, the health financing landscape changed in 2007 when Private Health Expenditure (PvtHE) surpassed General Government Health Expenditure. PvtHE is predominately out of pocket (OOP) with only 3.4% related to premiums for private insurance. In 2014, Household OOP Expenditure on health accounted for 52.8% of total health expenditure. OOP is known to be regressive and to impact negatively on households’ living standards.

**Objectives:**

This paper aims to examine trends in OOP in Mauritius, to assess its impacts through an analysis of key indicators of financial protection, namely catastrophic health expenditure (CHE) and impoverishment due to OOP health expenditure. It also aims to predict core determinants of CHEs.

**Methods:**

Household Budget Surveys (HBS) of 2001/2002, 2006/2007 and 2012 were the primary source data. CHE and impoverishment were used to assess financial hardships resulting from OOP health payments. The incidence of CHE was estimated at three threshold levels (10,25 and 40%), using the budget share and the capacity to pay approaches. Impoverishment due to OOP was measured by changes in the incidence of poverty and intensity of poverty using the US$ 3.1 international poverty line. Logistic regression analysis was used to identify determinants of CHE.

**Findings:**

Household CHE increased from 5.78% in 2001/02 to 8.85% in 2012 and 0.61% in 2001/02 to 1.25% in 2012, for 10 and 40% thresholds, respectively. The incidence of CHE was significantly higher in urban areas compared to rural areas. The highest levels of CHEs were among households’ heads, who are retired rising from 1.62% in 2001/02 to 3.71% in 2012, followed by households’ head who are widowed from 2.29% in 2001/02 to 2.63% in 2012 and homemakers from 2.12% in 2001/02 to 2.57% in 2012 at the 40% threshold. The share of households pushed below the poverty line due to OOP dropped from 0.4% in 2001/02 to 0.2% in 2006/07 before rising to 0.34% in 2012. In 2012, poverty gap occurred only among households under poorest quintile 1 (0.24%) and quintile 2 (0.03%). Overall poverty gap dropped from 0.08% in 2001/02 to 0.05% in 2012. Logistic regression analysis revealed that the odds ratio of facing CHE were significant only among households with heads being retired and with a presence of an elderly member in the household.

**Conclusion:**

Despite the rise in incidence of CHE between 2001 and 2012 the impact of OOP on the level of impoverishment and poverty gap has not been significant.

## Background

In 2015, the United Nations General Assembly adopted the 2030 Agenda for Sustainable Development [1]. The Agenda, which comprises 17 Sustainable Development Goals (SDGs) to be achieved by 2030, accentuates the importance for strengthened inclusive and integrated approaches to ensure that “no one is left behind” in achieving universal health coverage (UHC). The goal of UHC is to ensure that every individual and community, irrespective of their circumstances, is financially protected and receives the gamut of quality health services they require without running the consequences of financial hardship. The World Health Organization (WHO) and World Bank jointly developed a framework for tracking country and global progress towards UHC [2]. UHC is monitored within the framework of the SDGs though three dimensions, including coverage of essential health services and financial risk protection. A core indicator of financial risk protection is the proportion of population experiencing catastrophic health expenditure (CHE) due to out-of-pocket (OOP) heath payments. Impoverishment due to OOP health payments for health service is not an official SDG indicator but it is an important reference as it links UHC to the first goal of the 2030 agenda for sustainable development [2–4]. A distinctive feature of financial risk protection within the pursuit of UHC is that it provides the interface between the health systems and core dimensions of well-being [5].

Achieving financial protection from risks associated with household OOP expenditure on health care is today a core component of national health strategies in several middle-income countries, including Mauritius. WHO recognizes provision of financial risk protection as one criterion of good performance for health systems. Financial risk protection reflects the trade-off between on the one hand paying for health services that are needed and on the other hand paying for other basic needs, including education, food and housing [6, 7]. Mitigating financial risks is a core objective of universal access to health as high OOP expenditure on health can lead to households facing catastrophic health payments with expenditures exceeding a significant fraction of total household expenditures and, ultimately, leading to impoverishment, with households choosing to borrow money or selling assets to cater for health [8–11].

In 2010, 808 million people, representing 11.7% of the world’s population, experienced catastrophic spending with OOP payments on health exceeding 10% of total household consumption or income. It is estimated that at the 25% threshold of total household consumption or income, 179 million people incurred such payments and accounted for 2.6% of the world’s population. The incidence of catastrophic health spending in the African Region was lower than the global average in 2010 (11.4% in Africa versus 11.7% worldwide at the 10% threshold and 2.6% in Africa versus 2.5% worldwide at the 25% threshold). Further, at a US$ 1.90-a-day poverty line, an estimated 97 million people, representing 1.4% of the world’s population were impoverished due to OOP health payments. At the two international poverty lines (US $ 1.90-a-day and US $ 3.10 a-day) impoverishment rates in upper-middle-income countries are almost zero [2].

Mauritius is an upper middle-income country with a population of 1.26 million and per capita Gross Domestic Product (GDP) of US$ 9627 in 2016. Notwithstanding that GDP growth slowed down from an average of 4.7% (2000–2009) to 3.8% (2010–2016), the national economy has ably sustained social protection systems [12]. Mauritius is currently facing the demographic challenge of an ageing population coupled with increasing life expectancy and declining fertility rate below replacement level. The average life expectancy was 74.4 years in 2016 [13]. Poverty incidence based on World Bank $2 (PPP) a day poverty lines, dropped from 2.5% in 2001/02 to less than 2% in 2012 [14].

The health care system is a mix of public and private provision. Embracing principles of a welfare state, the country provides free health care provision in all government-owned health facilities. In 2014 72.8% of health care services (inpatient, outpatient and day care) were accessed through a network of public health facilities and the remaining 27.2% through privately owned health institutions [15].

In 2000 and 2006, General Government Health Expenditure (GGHE) accounted for 52 and 51.1% of Total Expenditure on Health (THE), respectively. In 2007 this trend was reversed with Private Health Expenditure (PvtHE) accounting for 51% of THE and out of pocket (OOP) expenditure on health representing 81.5% of PvtHE. National Health Accounts (2015) confirmed this trend as PvtHE accounted for 53.8% of THE and household OOP expenditure on health accounted for 98.2% of PvtHE in 2014. Consequently, household OPP expenditure on health exceeded GGHE by 16.3% in 2014 [16, 17].

As the bulk of household health payments are from current income (95.8%) and leaving a meagre share to health insurance reimbursements (3.4%), lack of financial protection is a challenge. About 8.1% of households borrowed from friends and relatives to meet their medical bills and another 3% of households were reported having to borrow from lending institutions [14].

With over 70% of the population accessing public health facilities and OOP expenditure on health outweighing GGHE, it is important to assess to what extent OOP payments for health services contribute to the lack of financial protection in Mauritius. As household OOPs expenditure on health and financial protection are negatively correlated it is equally important to explore and assess the incidence of catastrophic payments and impoverishment across different income strata of the population.

With a view to monitoring and assessing progress towards UHC in Mauritius and financial risk protection the scope of this study is two fold. First, we assess the incidence of Catastrophic Health Expenditure (CHE) at three commonly referred thresholds (10, 25 and 40%) and impoverishment due to OOP health expenditure. Secondly, we identify the main drivers of CHEs using Logistic Regression.

Two studies were carried out to determine the scale of CHE in Mauritius at 40% threshold. The World Health Survey estimated CHE incidence of 9% in 2003 while the Household Health OOP Survey carried out revealed a drop in the incidence of CHE in 2015 to 3.6% [6, 18]. In addition to the above surveys, the Global Monitoring Report of 2017 estimated that in 1996 the incidence of CHE for Mauritius at 6.79% (10% threshold) and 1.02% (25% threshold) [2]. The incidence of CHE in Mauritius is lower compared to the global average (9.7% at the 10% threshold and 1.9% at the 25% threshold in 2010) and Africa region (8.7% at the 10% threshold and 1.5% at the 25% threshold in 2010). However, the incidence of CHE in Mauritius is higher when compared with the European region, which represents at least 83% of countries with income per head equally or higher than Mauritius (0.3 and 0.1 percentage point, at the 10 and 25% thresholds, respectively) [2].

## Methods

### Sources of data

The Household Budget Surveys (HBS) of 2001/02, 2006/07 and 2012 were the primary sources data. This study used microdata from the three HBS conducted by Statistics Mauritius. Each HBS had a sample size of 6720 households and which were randomly selected. The sample comprised two separate samples, one of 6240 households (out of 325,000) for the main Island of Mauritius and another of 480 households (out of 10,000) for Rodrigues island. As the number of households in Rodrigues island was smaller, a larger sampling fraction was used to generate reliable estimates. Each sample was selected through a 2-stage design with probability proportional to size. At the first stage, clusters (comprising around 100 households) were selected with probability proportional to size of the population and followed at the second stage by random selection of households within these selected clusters [19].

Under the HBS, Household Expenditure is an aggregate of the twelve divisions of the UN Classification of individual consumption by purpose (COICOP), including health; transport; food [7]. However, transport expenses incurred to attend medical appointments and visits at health facilities are not accounted under the Health but instead under Transport.

### Data analysis

The two concepts related to financial hardships resulting from OOP payments or the absence of financial risk protection that will be addressed in this paper are CHE and impoverishment [5, 20].

CHE is encountered when the share of health costs exceeds household income or consumption expenditure at a given certain threshold. There is no universally agreed definition or threshold for assessing catastrophic health expenditure. However, the most commonly used threshold includes one at 40% of non-food expenditure (capacity to pay approach) and most recently both the 10 and 25% of the total household income (budget share approach) [21]. Several studies published in the recent years measured the incidence of CHE using both approaches to allow the sensitivity of the estimate using different approach [22–24].

In this paper the incidence of CHE is assessed using both standard approaches namely the capacity to pay and the budget share. This paper, also, assesses the sensitivity of findings to using the two measures. The dichotomy between the two approaches is that capacity to pay accounts for spending on necessities whereas that of budget share looks at total household income or expenditure [25].

CHE, under the capacity to pay approach, is household expenditure on health exceeding 40% of either expenditure less subsistence expenditure on food (i.e. total non-food expenditure), if food expenditure is less than subsistence spending; or total expenditure minus subsistence spending, if subsistence expenditure is greater than or equal to food expenditure. Average food expenditures of households with food shares in total expenditure from the 45th to 55th percentile was used as a proxy for subsistence food expenditure. For the budget share approach, two thresholds (10 and 25%) of total household income are used to assess CHE [26].

Impoverishment due to OOP is measured by changes in the incidence of poverty and the intensity of poverty before or after health spending. Changes in incidence of poverty is calculated as the difference in poverty headcount ratios due to household expenditure of OOP payments. The intensity of poverty is measured by the poverty gap ratio, which is the average amount by which total household expenditure falls short of the poverty line as a percentage of that line (counting the shortfall as zero for those above the poverty line) [27]. For the analysis an international poverty line of US$ 3.1 is chosen as this is more adapted to the national context given that Statistics Mauritius estimated a monthly poverty line of Rupees 13,310 for a standard household size of four members, representing approximately US$ 12.8 per household or about US$ 3.1 daily per individual [13].

To predict determinants and drivers of CHE, we conducted a logistic regression analysis. Prior to running the logistic regression, we assessed multicollinearity to rule out the presence of correlation between the explanatory variables. Furthermore, a post-estimation analysis was implemented to assess the model specification and the Hosmer and Lemeshow goodness-of-fit test was applied in this study. Stata v11.2 was used for data analysis of HBS.

## Results

### Participant’s characteristics

The majority of respondents were in the age group 15–59 years ranging from 65.9 to 66.2% for the three HBS survey years. Respondents were predominantly either married or single; ranging from 92.1% in 2001/02 to 90.2% in 2012. The proportion of economically active participants rose from 52.6% in 2001/02 to 57.6% in 2012 (Table 1).

**Table 1:** Demographic and socio-economic characteristics of households in the study subjects (*n* = 6720)

Variables	2001/2002 (%)	2006/2007 (%)	2012 (%)
Sex			
Male	49.7	49.4	48.9
Female	50.3	50.6	51.1
Age			
Under 5 years	7.8	7.4	5.4
5–14 years	17	16.3	14.8
15–59 years	66	65.9	66.2
60 years and above	9.2	10.4	13.6
Marital Status			
Married	45.7	46.6	47
Divorced / Separated /Widowed	7.9	8.8	9.8
Single	46.4	44.6	43.2
Activity Status (12 years & above)			
Currently active			
Employed	48	46.8	53.2
Without job but searching	4.6	4.4	4.4
Currently inactive			
Homemaker	23.5	22.9	20.8
Student	13.7	15.1	9.1
Disabled	2.2	2.0	2.3
Retired	7.0	7.2	9.9
Other	1.0	1.6	0.3
Household Size	3.9	3.7	3.5

### Catastrophic health Expenditure due to OOP payments by household characteristics

**Fig. 1:**
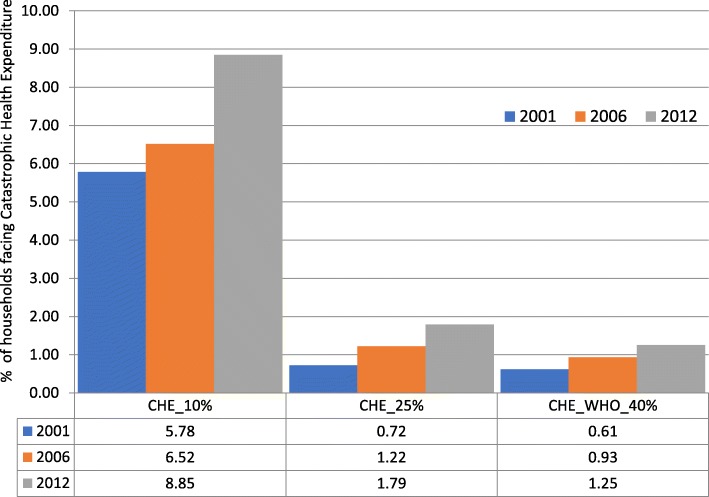
Household CHE at different thresholds, 2001–2012

Figure 1 shows that proportion of population facing CHE rose from 5.78% in 2001/02 to 8.85% in 2012 at threshold of 10% and from 0.61% in 2001/02 to 1.25% in 2012 at threshold of 40%.

Table 2 presents the social, demographic and economic characteristics of the head of households that experienced catastrophic expenditure across all three thresholds over the period 2001–2012. A general rising trend of CHE is consistent across all income quintiles and thresholds. Over the period 2001–2012, the incidence of CHE rose from 5.78 to 8.85% at threshold of 10%. In the same vein, the incidence of CHE rose from 0.61 to 1.25% at the threshold of 40%. In 2012, at the threshold of 10%, the incidence of CHE was 15.66% under the wealthiest quintile 5, against 3.24 and 5.46% for poorest income quintiles 1 and 2, respectively. For the same said year but at threshold of 40%, the incidence of CHE was 2.66% under quintile 5, against 0.51 and 1.01% for quintiles 1 and 2, respectively. Incidence of CHE in urban areas were higher than in rural areas across all thresholds and survey years, except in 2001/02 for thresholds of 25 and 40%. The urban rural gap has widened over the period 2001–2012 and across all thresholds.

**Table 2:** Incidence of CHE in relation to socio economic variables and income quintile, 2001–2012

		CHE_10% ^a^ (%)			CHE _25% ^b^ (%)			CHE_WHO_ 40% ^c^ (%)		
		2001/02	2006/07	2012	2001/02	2006/07	2012	2001/02	2006/07	2012
Quintile	Poorest Quintile 1	3.3	2.08	3.24	0.48	0.23	0.38	0.76	0.23	0.51
	Quintile2	4.84	2.95	5.46	0.38	0.28	0.68	0.36	0.57	1.1
	Quintile 3	5.18	6.95	8.25	0.28	1.81	1.17	0.36	1.35	1.06
	Quintile 4	6.56	8.24	11.64	0.92	1.21	1.98	0.62	0.72	0.95
	Wealthiest Quintile 5	9.05	12.37	15.66	1.52	2.58	4.74	0.96	1.79	2.66
Region	Rural	5.45	5.71	7.24	0.72	1.1	1.3	0.57	0.86	0.93
	(95% CI)	(4.7–6.1)	(4.9–6.4)	(6.4–8)	(0.4–0.9)	(0.8–1.4)	(0.9–1.6)	(0.3–0.8)	(0.5–0.11)	(0.6–1.2)
	Urban	6.3	7.7	11.39	0.71	1.36	2.6	0.67	1.04	1.77
	(95% CI)	(5.3–7.2)	(6.6–8.7)	(10.2–12.6)	(0.3–1.0)	(0.9–1.8)	(1.9–3.1)	(0.3–0.9)	(0.6–1.4)	(1.2–2.2)
Gender	Male	5.55	6.42	8.84	0.61	1.19	1.74	0.49	0.89	1.06
	(95% CI)	(4.9–6.1)	(5.7–7.0)	(8.0–9.6)	(0.4–0.8)	(0.9–1.4)	(1.3–2.0)	(0.3–0.6)	(0.6–1.1)	(0.7–1.3)
	Female	7.41	7.15	8.9	1.46	1.41	2.05	1.42	1.22	2.21
	(95% CI)	(5.8–8.9)	(5.6–8.6)	(7.4–10.3)	(0.7–2.1)	(0.7–2.0)	(1.3–2.7)	(0.7–2.1)	(0.6–1.8)	(1.4–2.9)
Marital	Married	5.48	6.4	8.83	0.56	1.17	1.64	0.46	0.92	1.02
	(95% CI)	(4.8–6.0)	(5.7–7.0)	(8.0–9.6)	(0.3–0.7)	(0.8–1.4)	(1.2–1.9)	(0.2–0.6)	(0.6–1.1)	(0.7–1.2)
	Widowed	7.51	8.45	10.12	2.04	1.41	2.81	1.75	1.19	2.63
	(95% CI)	(5.7–9.3)	(6.6–10.2)	(8.3–11.9)	(1.0–3.0)	(0.6–2.1)	(1.8–3.8)	(0.8–2.6)	(0.4–1.9)	(1.6–3.5)
	Divorced	11.12	4.35	10.15	2.78	0.79	2.05	3.89	0.4	1.76
	(95% CI)	(3.4–18.7)	(0.5–8.2)	(5.2–15.0)	(−1.2–6.7)	(− 0.8–2.4)	(− 0.2–4.3)	(−0.8–8.5)	(0.0–1.5)	(−0.3–3.9)
	Separated	5.37	1.47	4.23	0.55	0.49	1.23	0.55	0.16	1.32
	(95% CI)	(2.4–8.3)	(0.0–2.9)	(1.9–6.5)	(−0.4–1.5)	(− 0.0–1.3)	(0.0–2.4)	(− 0.4–1.5)	(0.0–0.6)	(0.0–2.6)
	Single	8.24	8.71	7.82	0.63	2.76	2.29	0.48	1.45	1.91
	(95% CI)	(4.9–11.5)	(5.5–11.8)	(4.7–10.8)	(−0.3–1.5)	(0.9–4.5)	(0.5–4.0)	(− 0.3–1.3)	(0.1–2.7)	(0.3–3.4.)
Education Level	Primary	5.4	5.34	7.23	0.85	1.05	1.22	0.81	0.77	1.26
	(95% CI)	(4.6–6.1)	(4.3–6.3)	(6.2–8.1)	(0.5–1.1)	(0.5–1.4)	(0.8–1.6)	(0.5–1.1)	(0.3–1.1)	(0.8–1.6)
	Secondary and above	6.01	7.35	10.05	0.53	1.42	2.15	0.36	1.02	1.0
	(95% CI)	(5.1–6.8)	(6.4–8.2)	(9.0–11.0)	(0.2–0.8)	(0.9–1.8)	(1.6–2.6)	(0.1–0.5)	(0.6–1.3)	(0.6–1.3)
Occupation	Employed	4.99	5.4	7.04	0.44	0.98	1.24	0.33	0.72	0.53
	(95% CI)	(4.3–5.5)	(4.7–6.0)	(6.3–7.7)	(0.2–0.6)	(0.7–1.2)	(0.9–1.5)	(0.1–0.4)	(0.4–0.9)	(0.3–0.7)
	Homemaker	7.7	7.5	8.33	2.57	1.95	2.5	2.12	1.66	2.57
	(95% CI)	(5.1–10.2)	(5.2–9.7)	(6.0–10.6)	(1.0–4.0)	(0.7–3.1)	(1.1–3.8)	(0.7–3.5)	(0.5–2.7)	(1.2–3.8)
	Student	–	–	7.69	–	–	7.69	–	–	7.69
	(95% CI)	–	–	(−22.4–37.8)	–	–	(−22.4–37.8)	–	–	(−22.4–37.8)
	Retired	9.18	12.3	16.37	1.59	2.32	3.91	1.62	1.79	3.71
	(95% CI)	(7.3–11.0)	(10.2–14.3)	(14.3–18.3)	(0.7–2.3)	(1.3–3.2)	(2.8–4.9)	(0.8–2.4)	(0.9–2.6)	(2.6–4.7)
	Unemployed	7.51	0.82	8.92	0.63	0.82	–	0.63	–	–
	(95% CI)	(0.0–14.9)	(−2.3–3.9)	(1.5–16.3)	(− 1.6–0.028)	(−2.3–3.9)	–	(− 1.6–2.8)	–	–
	Other	9.4	12.01	9.99	1.63	1.69	1.5	1.63	1.88	1.83
	(95% CI)	(4.7–14.0)	(7.2–16.8)	(5.6–14.3)	(− 0.3–3.6)	(−0.2–3.5)	(−0.2–3.2)	(−0.3–3.6)	(0.0–3.8)	(−0.1–3.7)
Total		5.78	6.52	8.85	0.72	1.22	1.79	0.61	0.93	1.25

^a^Expenditure is considered as being catastrophic if a household’s financial contributions to the health system exceed 10% of total household consumption expenditure or income

^b^Expenditure is considered as being catastrophic if a household’s financial contributions to the health system exceed 25% of total household consumption expenditure or income

^c^Expenditure is considered as being catastrophic if a household’s financial contributions to the health system exceed 40% of capacity to pay (expenditure minus subsistence expenditure on food, if food expenditure is less than subsistence spending, or total expenditure minus subsistence spending, if subsistence expenditure is greater than or equal to food expenditure)

Over the period 2001–2012 the increasing incidence of CHE were driven by households’ head who are retired (rising from 1.62% in 2001/02 to 3.71% in 2012), and followed by households’ head who are widowed (rising from 2.29% in 2001/02 to 2.63% in 2012) and homemakers (rising from 2.12% in 2001/02 to 2.57% in 2012) at the 40% threshold.

In 2012 at threshold of 25%, incidence of CHE among a married head of households is 1.02% as compared to 1.91% for a single headed household. In terms of gender, 2.21% of female headed households experienced CHE as compared to 1.06% among male headed households.

### Impoverishment due to OOP health spending

**Fig. 2:**
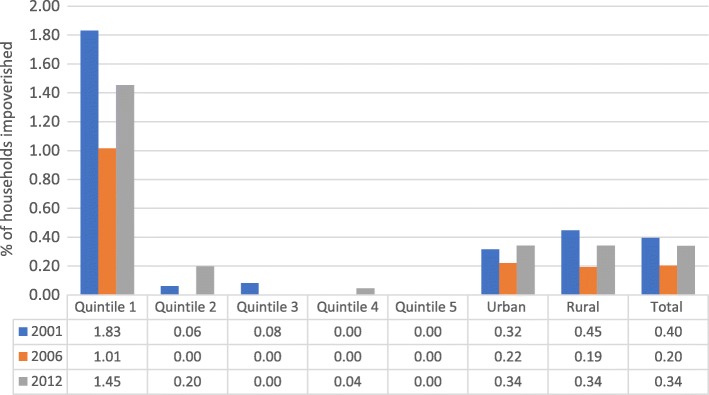
Impoverishment due to OOP based on International Poverty Line of US$ 3.1 daily

Figure 2 shows the level of impoverishment due to health expenditures by quintiles and region using the international poverty line of US$ 3.1 daily. As shown in Fig. 2, the incidence of impoverishment declined from 0.4% in 2001/02 to 0.2% in 2006/07 and rose to 0.34% in 2012. Between 2001/02 and 2006/07 impoverishment headcount dropped in both rural and urban areas. The drop in impoverishment headcount was higher in rural (0.26 percentage point) compared to urban (0.10 percentage point) areas. However, in 2012 impoverishment headcount was same in both rural and urban areas (0.34%).

### Poverty gaps

**Fig. 3:**
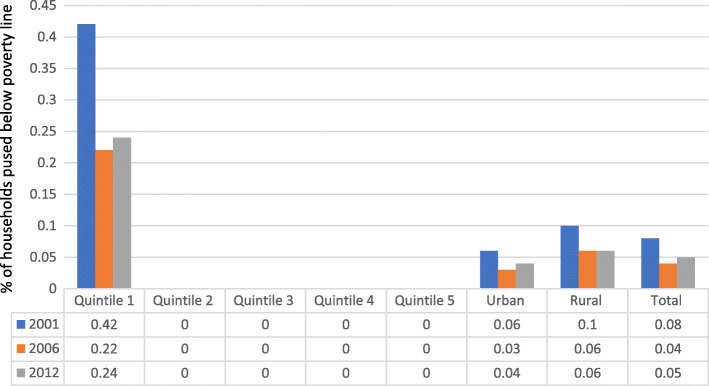
Poverty gap due to OOP based on International Poverty Line of US$ 3.1 daily

Figure 3 shows that poverty gap due to OOP health spending dropped from 0.08% in 2001/02 to 0.04% in 2006/07 before rising to 0.05% in 2012). Region wise, poverty gap due to OOP health spending in urban area maintained a general drop from 0.06% in 2001/02 to 0.04% in 2012. In the same vein, poverty gap in rural areas dropped from 0.1% in 2001/02 to 0.06% in 2012.

### Drivers of catastrophic health expenditure

The logistic regression results for determinants of CHE using three thresholds are shown in Table 3. The results indicate that across all three thresholds only two variables, household having one member with 60 years or over, and head of household being retired significantly contributed to CHE as their respective relative *p*-values are less than 5%. Widowed Head of Households experienced CHE at 90% significance level. Some other variables were significant determinants of CHE, with *p*-values less than 5%, for two threshold levels, including head of household being homemakers (CHE_25% and CHE_WHO_40%) and head of household having at least secondary education level (CHE_10% and CHE_25%).

**Table 3:** Logistic regression odds ratio (95% CI) of CHE for socio economic variable, 2001–2012

Variables	Reference	CHE_10%		CHE_25%		CHE_WHO_40%	
		*P*-value	Odds Ratio (95% CI)	*P*-value	Odds Ratio (95% CI)	*P*-value	Odds Ratio (95% CI)
Year 2006	(Yes = 1, No = 0)	0.349	1.082 (0.918–1.275)	0.017	1.622 (1.092–2.409)	0.107	1.420 (0.927–2.176)
Year 2012	(Yes = 1, No = 0)	0.000	1.446 (1.245–1.680)	0.000	2.046 (1.413–2.962)	0.043	1.507 (1.013–2.241)
Gender of household head as being female	(Yes = 1, No = 0)	0.475	1.103 (0.843–1.442)	0.364	0.756 (0.414–1.382)	0.368	1.339 (0.709–2.529)
Region as being urban	(Yes = 1, No = 0)	0.000	1.297 (1.143–1.471)	0.228	1.189 (0.898–1.575)	0.254	1.207 (0.874–1.667)
Education level of head of household being secondary and above	(Yes = 1, No = 0)	0.000	1.509 (1.322–1.722)	0.000	1.750 (1.324–2.313)	0.238	1.214 (0.880–1.674)
Head of household being widowed	(Yes = 1, No = 0)	0.143	0.813 (0.615–1.073)	0.101	1.685 (0.904–3.140)	0.676	1.154 (0.589–2.263)
Head of household being divorced	(Yes = 1, No = 0)	0.893	1.035 (0.626–1.712)	0.391	1.576 (0.557–4.461)	0.519	1.456 (0.464–4.567)
Head of household being separated	(Yes = 1, No = 0)	0.018	0.565 (0.352–0.907)	0.932	0.958 (0.356–2.575)	0.637	0.753 (0.231–2.452)
Head of household being single	(Yes = 1, No = 0)	0.704	1.060 (0.784–1.433)	0.321	1.399 (0.721–2.717)	0.736	1.136 (0.541–2.389)
Presence of at least one child of less than 5 years in household	(Yes = 1, No = 0)	0.501	1.054 (0.904–1.229)	0.330	0.832 (0.574–1.205)	0.840	0.958 (0.629–1.458)
Presence of at least one elderly (> 60 years) member in the household	(Yes = 1, No = 0)	0.000	1.522 (1.295–1.789)	0.008	1.606 (1.133–2.277)	0.006	1.756 (1.179–2.615)
Head of household being a homemaker	(Yes = 1, No = 0)	0.124	1.299 (0.931–1.812)	0.031	2.058 (1.069–3.963)	0.006	2.594 (1.318–5.105)
Head of household being retired	(Yes = 1, No = 0)	0.000	1.989 (1.648–2.400)	0.000	2.444 (1.660–3.597)	0.000	3.389 (2.226–5.158)
Head of household being unemployed	(Yes = 1, No = 0)	0.788	1.116 (0.501–2.486)	0.386	0.535 (0.130–2.200)	0.424	0.445 (0.061–3.235)
Head of household having other activity	(Yes = 1, No = 0)	0.003	1.749 (1.214–2.520)	0.060	2.088 (0.970–4.497)	0.000	3.627 (1.809–7.274)

Catastrophic Health Expenditure was taken as dependent variable whereas others taken as independent variables. Significant at *p*-value < 0.05 levels, CI = Confidence Interval

At the 40% threshold the odds ratio depicts that the likelihood of CHE was 3.39 fold higher among households whose head is a retiree, followed by household whose head is a homemaker and has a member with 60 years, with the likelihood of CHE estimated at 2.59 fold and 1.76 fold, respectively. Other variables such as gender, residence, marital status, presence of a child of less than 5 years in the household and employment status of household head were not significant drivers of CHE.

Logistic regression and odds ratio to determine drivers for impoverishment for socio economic variables was also carried out. However, none of the socioeconomic variables considered as drivers of CHE were significant as the *p*-values are over 10%.

## Discussion

The Household Health OOP Survey carried out revealed that incidence of CHE in 2015 was 3.6% [13] as compared to 1.25% in this paper for 2012. The difference in estimates of incidence is due to distinct sample. This paper is based on sample drawn from the HBS of Statistics Mauritius while the 2015 Household Health OOP Survey has a different sample.

The incidence of CHE has maintained an upward trend over the period 2001–2012, rising by 1.07 percentage point at the 25% threshold level. Even with such low incidences of catastrophic payment, it can be inferred that financial risk protection is a challenge in Mauritius. Increasing incidence of catastrophic payment may be mainly attributed to a general rise in the real disposable income and improved living standards coupled with increasing expectations for less waiting time, more patient-centred care resulting with more patients turning to medical treatment in the private health sector [6, 20].

Compared to South Africa, which is also an upper middle-income country with health services provision driven by the public sector and similar level of UHC service coverage index, the incidence of CHE is relatively higher in Mauritius (0.93% in 2006/07 versus 0.09% in 2006/07 at 40% threshold) [28]. Moreover, this paper confirms progress made in Mauritius to lower incidence of impoverishment due to OOP health payments. Whereas a survey of 122 countries concluded a rising incidence of impoverishment over the period 2000–2010 at the US$ 3.10 a day poverty-line, the trend for Mauritius has been on the decline from 2001/02 to 2012, albeit a minor rise in 2006/07 [29].

The rapid epidemiological transition characterised by rising prevalence of chronic diseases and metabolic syndromes, in particular Diabetes since the early 1980’s, coupled with a rapidly ageing population have largely fuelled household OOP Payments and worsened incidence of CHE [30].

Since 2001 a grant within the range of Rupees 500,000 to 800,000 (approx. from US$ 15,125 to 24,200) is available for patients resorting to treatment outside Mauritius. Additionally, foreign medical teams are called to perform complicated surgeries in public hospitals and patients have no fees to pay. These policy measures partly explain the relatively low incidence of CHE and impoverishment in the lower quintiles compared to other countries.

As opposed to other countries, this study shows that the urban population faces higher incidence of CHE than the rural population. The three HBS revealed that the share of health expenditure of total household expenditure is systematically higher in urban areas (5.3% in 2012) than in rural areas (3.7% in 2012). Furthermore, as the household average disposable income is at least 20% higher among the urban population, the rural population is less able to afford healthcare from fee paying private institution and prefer to reserve their income on essential needs and seek healthcare from public facilities at no cost. In the same breadth progress made towards promoting equitable access to public health care facilities in rural areas, with primary health centres located within a radius of 5 km from any residential areas and three of the five main regional public hospitals located in the rural districts, have ensured that the rise in the incidence of CHE in rural areas is lower than that in urban areas.

In 2014 National Health Accounts revealed that household OOP expenditure on health in the private represented 51% of THE but on the other hand only 27.2% of health services were accessed through private network. This is largely explained by the fact that the major driver of OOP payments on health by households in 2014 were pharmaceutical products (27.07%) followed by medical supplies and disposables (20.28%) [15]. There is a misconception that brand name medicines are more effective than generic ones [31, 32]. In Mauritius, instead of making good use of generic medicines from public health facilities, substantial number of households prefer to pay for brand name medicines [33, 34]. Patients often resort to self-medication as a form of therapy and consequently undertake hefty purchases of medicine products over the counter. These have important cost implications for the household as well as on the incidence of CHE. A survey on Medicine Prices in Mauritius informed that in the private sector the cheapest generic medicines on sale were priced nearly 6 times their international reference price. Furthermore, originator brand medicines were priced at nearly 20 times their international reference price. On average the survey estimated that originator brand premium of medicines is at least three fold costlier compared to the lowest generics medicine products in the private sector [29]. Conversely, the public sector procures generic products, at a cost about 34% less than the international reference prices.

The policy announcement made to consider the introduction of a voluntary health insurance scheme for public employees, where the Government will pay 50% of premium in favour of civil servants, is expected in the future to improve financial risk protection coverage for at least some 30,000 civil servants when seeking medical care in the private sector. Though this measure will not impact directly on the poor, but as more civil servants resort to medical care privately, public hospitals will be less crowded and waiting time at hospital level will be reduced. Long waiting time is a major factor discouraging the poor to attend public hospitals as they often absent from work and forego their daily pay. Reducing the bottleneck at public hospitals will incite the poor population to seek treatment in public facilities. Thus, avoiding the likelihood of catastrophic payments. Furthermore, it is recommended that the tax reliefs on health insurance policies for income tax purposes be increased to promote financial risk protection for households.

The regulatory framework for private health sector has been lacking and this resulting in price differentials of health interventions from one health facility to the other. The absence of a harmonised rate across private health facilities and asymmetric information of the rates imposed have contributed in rising catastrophic payments and impoverishment resulting from OOP payments. A harmonisation of rates for in patients and out patients for common pathologies in private hospitals and clinics will contribute towards lowering incidence of catastrophic payments and impoverishment resulting from OOP health expenditure.

Ensuring the monitoring of financial risk protection as part of the SDG reporting framework requires reliable and periodic household surveys that contain information on health-specific and other expenditures. While household budget surveys have been institutionalised and are conducted every 5 years in Mauritius, an important lacuna, however, is that not much provision is made for analysis of health expenditures and utilisation of health services in both public and private sector.

The results obtained from the logistic regression confirmed the findings of other studies done that socioeconomic variables have a significant impact on CHE. The likelihood of been faced with CHE was between 1.22 and 1.76 times more among household which has a member over 60 years of age. Likewise, the same conclusion could be inferred among household where the head is a retiree as the risk varied between 2 and 3.4. These two independent variables which were significant when determining CHE over the period 2001–2012 would pose a major challenge in the medium-long perspective with a rapid ageing of the population.

An important limitation of the analysis is that individuals who do not seek care due to various barriers, such as geographical inaccessibility, stigmatisation, are not captured in the estimates of CHE. Thus, underestimating incidence of catastrophic payments and impoverishment. Another constraint is that component of reimbursement of health payments has not been deducted from Health Expenditure at household level as the HBSs do not differentiate between the different types of insurance reimbursement.

## Conclusion

Though CHE has been on the rise across most income groups over the three consecutive HBS period the impact on the level of impoverishment and poverty gap has been low. A caveat when interpreting low incidence of CHE and impoverishment is that this could well be due that segment of the population is not receiving health care they need as they cannot access or afford same in the first instance. This is substantiated by the UHC Service Coverage Index, which is the average coverage of essential health services (such as reproductive, maternal, new-born and child health, infectious and noncommunicable diseases), estimated at only 64 for Mauritius in 2015 [2]. Nevertheless, these estimates provide important policy guidance on the trends in financial protection as well as identifying population groups that are prone to financial risk and therefore warranting urgent policy action to remedy them. Moreover, a comprehensive Benefit Incident Analysis is recommended to determine the overall distributional impact and benefits of free health services on the poor versus the rich.

## Abbreviations

CHE: Catastrophic Health Expenditure
CI: Confidence Interval
GGHE: General Government Health Expenditure
HBS: Household Budget Survey
OOP: Out of Pocket Payments
PvtHE: Private Health Expenditure
SDG: Sustainable Development Goal
THE: Total Expenditure on Health
UHC: Universal Health Coverage
WHO: World Health Organization
